# Differentially expressed and alternately spliced genes as a novel tool for genotoxicity: a computerized study in ATT-myc transgenic mice for the recognition of genotoxic and non-genotoxic chemical

**DOI:** 10.3389/fgene.2025.1505379

**Published:** 2025-03-28

**Authors:** Mansour A. Alghamdi, Eman M. El Nashar, Mahmoud Elalfy, Norah S. Al-Zahrani, Mohammed A. Alshehri, Mohammad El-Nablaway, Khulood M. Al-Khater, Rashid A. Aldahhan, Eman G. El-Hadidy, Fathy Sleem, Ahmed Aljazzar, Jürgen Borlak, Mona Elhadidy

**Affiliations:** ^1^ Department of Anatomy, College of Medicine, King Khalid University, Abha, Saudi Arabia; ^2^ Genomics and Personalized Medicine Unit, The Center for Medical and Health Research, King Khalid University, Abha, Saudi Arabia; ^3^ Clinical Science Department, College of Veterinary Medicine, King Faisal University, Al Hofuf, Saudi Arabia; ^4^ Forensic and Toxicology Department, Faculty of Veterinary Medicine, Mansoura University, Mansoura, Egypt; ^5^ Department of Clinical Biochemistry, College of Medicine, King Khalid University, Abha, Saudi Arabia; ^6^ Department of Child Health, College of Medicine, King Khalid University, Abha, Saudi Arabia; ^7^ Department of Basic Medical Sciences, College of Medicine, AlMaarefa University, Riyadh, Saudi Arabia; ^8^ Department of Medical Biochemistry, Faculty of Medicine, Mansoura University, Mansoura, Egypt; ^9^ Department of Anatomy, College of Medicine, Imam Abdulrahman Bin Faisal University, Dammam, Saudi Arabia; ^10^ Mathematics and Bioinformatics Department, Faculty of Science, Damietta University, Damietta, Egypt; ^11^ Pathology Department, College of Veterinary medicine, King Faisal University, Al Hofuf, Saudi Arabia; ^12^ Centre for Pharmacology and Toxicology, Hannover, Germany; ^13^ Department of Medical Physiology, Faculty of Medicine, Mansoura University, Mansoura, Egypt; ^14^ Department of Medical Physiology, Faculty of Medicine, Al-Baha University, Al Baha, Saudi Arabia

**Keywords:** gene expression pathways, exon array, att-myc model, HCC, genotoxic, non-genotoxic, NDEA, and BHT

## Abstract

**Background:**

Transgenic mice and gene expression in analyses were employed to evaluate hazardous chemicals.

**Methods:**

Mice received weekly doses of NDEA (75 mg/kg) for six weeks and twice-weekly doses of BHT (300 mg/kg) for eight weeks. Gene expression and splicing alterations in the livers of six transgenic mice for each treatment of NDEA and BHT were examined using the MouseExon10ST array.

**Results:**

Six hybridizations revealed 645 genes with significant expression changes, and 181 genes showed both expression and splicing alterations (p < 0.01). Furthermore, 2021 genes demonstrated significant exon–group interactions, indicating potential alternative splicing. Pathway analysis identified enriched groups in GOMolFn, GOProcess, GOCellLoc, and Pathway classes, with a higher representation of alternatively spliced and expressed genes (p < 0.01).

**Discussion:**

Among the top expressed genes was TAT, which encodes the mitochondrial enzyme tyrosine aminotransferase, involved in tyrosine metabolism and recognized as a novel tumor suppressor gene linked to hepatocellular carcinoma (HCC). Additionally, HNF-4, a transcription factor, plays a crucial role in TAT expression.

**Conclusions:**

This method can be used to identify genotoxic compounds in the att-myc model for short-term toxicity.

## Introduction

Genotoxicity is the ability to damage genetic information within a cell, resulting in mutations that may lead to malignancies (Hartwig et al., 2020). Genotoxic substances induce damage to the genetic material in the cells through interactions with the DNA sequence and structure (Jacobs et al., 2016). Various tests for genotoxicity, such as chromosomal aberrations, micronuclei, and sister chromatid exchanges, have been employed in both acute and chronic studies (Ames et al., 1973). On the other hand, non-genotoxic carcinogens promote cancer through processes that do n't involve mutations, such as hormonal influences, cell damage, increased cell growth, or epigenetic alterations (Desaulniers et al., 2021; Choy and Assessment, 2001). Additionally, genotoxicity tests typically include cytogenetic assays to assess significant DNA damage. Single- and double-strand DNA breaks are key alterations that can lead to mutations. These breaks can be detected by observing the formation of foci of histone H2Ax-gamma, which is a marker of DNA damage (Rahmanian et al., 2021).

The failure, downregulation, or mutation of gene repair mechanisms, along with epigenetic changes (Chen et al., 2020)such as DNA methylation, specific histone methylation or acetylation (Rajan et al., 2020), and DNA damage induced by oxidative stress (MJCl, 2012), all contribute to tumor development.

Microarray-based genomics using a short-term *in vivo* model were deemed a fast and superior method for characterizing carcinogens through statistical and mechanistic analyses. A previous study included differentially expressed genes and associated pathways in cellular processes, uncovering significant mechanisms involving key cellular components (Lee et al., 2013).

In toxicological studies, the gene expression and bionformatics analysis offers new insights for testing chemicals and identifying pathways potentially linked to cancer, although these pathways often require further validation to develop new cancer biomarkers or drug therapies. The specificity of identified genes should be closely related to cancer development. Bioinformatics is applied in both *in vitro* and *in vivo* models, and both require additional validation (Zhao et al., 2012). Additionally, when the using cDNA microarrays and the gene expression profiles improved the differentiation between genotoxic and non-genotoxic effects for 20 chemical carcinogens in HepG2 cells (Van Delft et al., 2004; Lee et al., 2014).

Previous research found that genotoxic (GTX) carcinogens activate the p53 tumor suppressor gene, leading to cell cycle arrest, apoptosis, and DNA repair processes. This activation regulates multiple genes, including Cdkn1a, Mdm2, and Bcl2 (Ellinger-Ziegelbauer et al., 2004). Additionally, a selection of 100 genes was identified to differentiate between genotoxic and non-genotoxic hepatocarcinogens. Differential gene expression induced by chemicals was examined using DNA microarrays and validated through quantitative real-time PCR (Furihata et al., 2016).

 ATT-myc model of liver tumor was first identified by Dalemans et al. (1990) and we choices it for current study as it had 1 year displayed only liver dysplasia and HCC appeared later after 12 months a. Notably, similar record reported that ATT-myc model displayed HCC later after 12 months (Santoni-Rugiu et al., 1996) and so this model favor the testing of chemicals as well as known carcinogens, DEN, but it need further validation (Hueper et al., 2012) for testing of chemical as Rash2 transgenic model and p53−/− transgenic mice.

The goal of this study is to identify gene classifiers through bioinformatics analysis that can differentiate between genotoxic carcinogens (like diethyl nitrosamine) and non-genotoxic carcinogens (such as butylated hydroxytoluene), using ATT-myc transgenic mice and liver gene expression profiles.

## Materials and methods

This study involved 72 transgenic ATT-Myc mice of both sexes and 12 non-transgenic mice. All animals were housed in cages with 1 to 4 mice each on sawdust bedding and under a 12-h light–dark cycle with 50% relative humidity and a temperature of 22°C. They were provided with a standardized diet and had free access to water (Zucht, ssniff M-/10 mm, complete mice diet, ssniff Requirements GmbH, DE-59494, www.ssniff.de). This study was approved by Ethical Committee of king Faisal university (KFU-25-ETHIC53114) and the city of Hannover, Germany (AZ:.33.9-42502-04-08/1619).

### Study design and treatment

The mice were categorized into three groups; the first and second groups each consisted of 48 transgenic mice, with 12 males and 12 females in each group. The third group included 24 non-transgenic mice of both sexes as a vehicle control. The first group of transgenic mice received NDEA (99% purity, Sigma Aldrich, Germany) at a dose of 100 mg once per week for 6 weeks, starting at 2 months of age. The second group of transgenic mice received BHT at a dose of 300 mg/kg twice per week for 8 weeks. Both transgenic and non-transgenic control mice were used for comparison. During the six-week period, transgenic mice were administered a saline solution containing 100 mg/g NDEA weekly, while the control mice received only saline injections.

### Sample collection and preparation

Mice were anesthetized with CO2, and their thoraxes were opened using standard surgical techniques. The liver was then extracted using PBS at the end of the treatment period at 4 months of age. The liver tissue was promptly frozen in liquid nitrogen and stored at −80°C.

### Isolation through hybridization and RNA

Total RNA was extracted from frozen liver tissues using the RNeasy total RNA isolation protocol from QIAGEN. The process adhered strictly to the Target Labeling Assay Manual. This included ribosomal RNA reduction, cDNA synthesis, cRNA hydrolysis, fragmentation, terminal labeling, hybridization, washing, chip staining, GeneChip scanning, and data interpretation.

### Data analysis, normalization, and comparison

**FIGURE 1:**
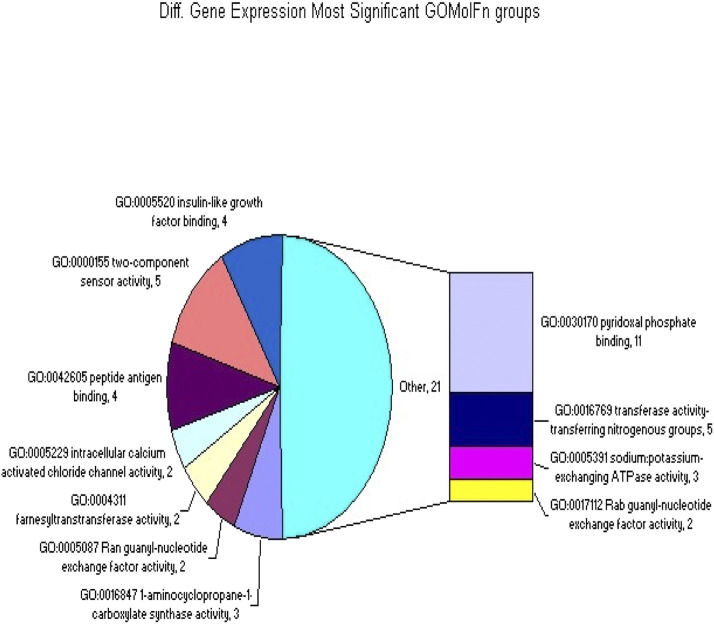
The GOMolFn gene classifications that were significantly overrepresented in the set of differentially spliced or expressed genes.

**FIGURE 2:**
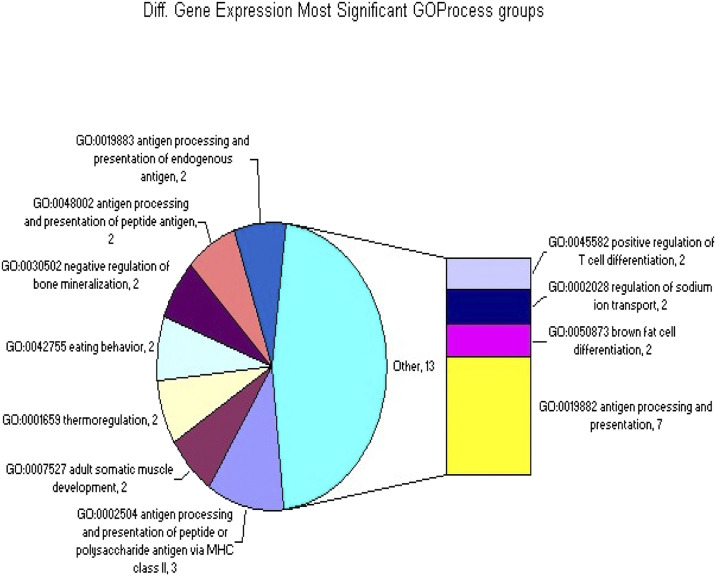
The GOProcess gene classifications that were significantly overrepresented in the set of 414 differentially spliced or expressed genes.

The analysis was conducted using a mixed-model analysis of variance on 6 hybridizations of NDEA treatments and 6 hybridizations of BHT-treated transgenic samples, and all were processed on the MouseExon10ST array. The data were analyzed using the XRAY (version 3.2) software on 15 December 2010. Gene expression for both probes was normalized against historical data (Figures 1, 2). Fold changes were deemed significant with a p-value of ≤0.05, and statistical tests were performed using the Student’s t-test.

### Array normalization

The input files were normalized using complete quantile normalization (Bolstad et al., 2003). For each probe expression value in each input array, the average of all array points was used instead of the array percentile probe value.

### Low-level data handling

The 4,549,897 probes were subsequently converted into analytical values, as described below. Probes with a GC count greater than 17 or less than 6 were excluded from the analysis. The remaining probe scores were then transformed by multiplying them by the logarithm of 0.1.

### Background error

In exon arrays, individual mismatch probes are not used. Instead, a set of specially designed probes are employed to provide context. The MouseExon10ST antigenomic. bgp file included a list of background probes, which were categorized by their CG content. BGP files are also available for download at www.affymetrix.com. To adjust for the background, the median expression score of background probes with the same GC content was subtracted from each probe score.

### Probe set expression scores

The MouseExon10ST array featured 1,185,965 probe sets, which were generally groups of four probes, though this was not always the case.

### Probe set expression scores and annotation filtering

The expression score for a probe–probe set was calculated by taking the median of its expression scores. Probe sets with fewer than three probes that passed all relevant tests were excluded from the analysis. In the exon arrays, the reliability of individual probes and probe sets was dependent on the accuracy of their genomic annotations. The probe sets were classified into three reliability levels: Core, Extended, and Complete, with Core being the most reliable. For example, “Core” probe sets were linked to high-quality genomic features, such as RefSeq or Ensembl transcripts, while “Extended” and “Complete” probe sets were associated with less reliable annotations, such as gene prediction algorithms and EST hits. Only “Core” probe sets were used in this study.

### Probe set presence/absence and the removal of non-expressed probe sets

Alternative splicing tests might produce false positives if non-expressed probes lead to “non-parallel” expression patterns across the genome. A probe set in a given category was deemed more expressed in the context only if the integral from T0 to infinity of the standard normal distribution was less than or equal to 0.001. Here, Group Size refers to the number of CEL files in the group; T0 was calculated as.

$$Sqr\left(GroupSize\right)\times \left(T-P\right)Sqr\left(Pvar\right)\backslash text\left\{Sqr\left(GroupSize\right)\right\}\backslash times\backslash frac\left\{\left(T-P\right)\right\}\left\{\backslash text\left\{Sqr\backslash \left(Pvar\right)\right\}\right\}Sqr\left(GroupSize\right)\times Sqr\left(Pvar\right)\left(T-P\right)$$

P represents the average of background probe values adjusted for GC content; Pvar is the total variance of background probes divided by the square of the number of probes in the probe set.

To determine if the average expression of background probes with a similar GC content to the probe set was higher than that of the background probes used as a reference for the probe set, we first calculated the dispersion from the variance of the background probe set.

### Filtering invariant probe sets

Probe sets with low variance were removed from the analysis using a Chi-square test. A probe set was considered to have low variance if its transformed variance fell below the 90 percent confidence interval of the Chi-squared distribution with (N-1) degrees of freedom. This was determined by comparing the variance of the gene probe set to the Chi-squared value for (N-1) degrees of freedom.

Here, NNN represents the number of input CEL files, (N−1)(N-1)(N−1) denotes the degrees of freedom for the Chi-squared distribution, and “probe set variance for the gene” refers to the average variance of probe sets associated with the gene. While this method is generally effective, it is important to note that probe sets, and probe sets across genes are not independent. Additionally, we compared the variance of the probe set with a random variable, whereas the Chi-square test of variance is typically used to compare a variance against a constant value.

The filtering results are summarized in the following table.

(*) clusters of transcripts that contain four to two hundred passing probe sets.

(**) At least three passing probes are present in a probe set.

The following Table 1. summarizes the results of filtering.

**TABLE 1:** The table summarizes the results of filtering.

Filtering step	Filter	Probes	Probe sets	Transcript Clusters
0	Total on Chip	4,549,897	1,185,965	270,096
1	Core Probe Sets	839,727	218,187	15,701 (*)
2	Pass Filter 1 and Probes with GC Counts between 6 and 17	800,357	201,370 (**)	
3	Pass Filters 1 and 2 andProbe Sets Expressed Above the Background	390,024	98,077 (**)	
4	No Absolute Score Filter Used	390,024	98,077 (**)	
5	Pass Filters 1, 2 3, and 4 and Pass the Background	348,730	87,705 (**)	7,864 (*)

We examined 7,864 genes for differences in gene expression or alternative splicing patterns between the classes. Due to the filtering of probe set annotation levels (Core) and the exclusion of probe sets not expressed in the bht-1-mf and ndea-1-mf groups, the number of remaining transcript clusters with more than four probe sets was reduced. As a result, the number of genes tested may have been considerably lower than the total number of transcript clusters on the chip.

### Identification of group-specific gene expression and alternative splicing

To find genes with group-unique gene expression or alternative splicing, the researchers used a mixed-model nested study of variance (Montgomery, 2017). The nested model was suitable because expression data points were obtained in batches based on hybridizations rather than by randomly sampling data or individual CEL files. A mixed model was employed because the CEL files were treated as random variables—our focus was on the effects of multiple arrays rather than individual CEL files. Groups and exons had a continuous effect. If the experiment were repeated, the designation of states would be either random or fixed. Analysis of variance (ANOVA) was applied to the data according to a linear model.

### Multiple-test correction

For each gene tested, the probability of a “false positive” (or “Type I Error”) was 0.01 under ideal randomization conditions. However, this significance value could be misleading because we were testing a large number of “independent” genes, and the chance of finding a false positive increased as the number of genes tested grew (Benjamini and Hochberg, 1995). To address this issue, we used the Benjamini and Hochberg false discovery rate (FDR) method, which was initially introduced by Simes (Dalemans et al., 1990). This method controls the family-wide error rate in a weaker sense by managing the expected proportion of false positives (unlike methods such as Bonferroni correction, which controls false discoveries in a stronger sense by bounding the probability of any false positives; however, such methods can be overly conservative and have reduced power for these types of studies) (Benjamini and Hochberg, 1995) and originally proposed by Simes (Simes, 1986).

For the third-largest p-value and on, NNN represents the total number of genes analyzed. Next, genes with a corrected p-value greater than RRR could be excluded to determine the project’s false discovery rate (FDR), where RRR was the threshold. Alternatively, the FDR could be set to the highest corrected p-value, allowing all significant results before correction to be retained. While this method is commonly used for expression analysis, it operates under the assumption that individual tests are independent, which prevents it from capturing gene interactions.

### Determining tissue presence/absence using group expression above background

To determine if a gene was expressed in a group (or tissue), we calculated the p-value to test the null hypothesis that the average expression of CEL files from that group did not exceed the background level. In other words, the p-value indicated the probability of observing the gene expression level under the null hypothesis, which assumed that tissue expression was no greater than the background level. If the p-value was below the significance threshold of 0.01 and the null hypothesis was rejected, we could conclude that the gene was likely expressed in the tissue. The assessment of [1 – Cumulative Standard Normal (N0) ] at N0 = Sum (probe–GC Background)/Var yielded this significance given the probes in a given gene. Here, Var represents the average background variance, GC Background is the median score at a given probe GC count, and the sum is over all probe scores belonging to CEL files in the group.

### Group expression level filters to reduce false positives in alternative splicing

Significant variations in a gene’s expression levels across tissues can cause nonlinear behaviors that deviate from the expected expression model, potentially leading to false positives for alternative splicing. An example of “non-parallel” expression between groups occurs when exon expression in one group approaches the background levels (or saturation) while other groups remain within the dynamic range. This happens because expression values in the dynamic range are more flexible, whereas those near the background or saturation are “dampened”. Such non-parallel behavior might be incorrectly interpreted as group-specific alternative exon usage. To address this issue, we used p-values for group expression of the gene to identify and exclude these cases.

### Only one group expressed

#### Data analysis, normalization, and comparison

This study utilized a mixed model analysis of variance to examine six hybridizations of NDEA treatments and six hybridizations of BHT samples using the MouseExon10ST array. The analysis was conducted by the installer with XRAY (version 3.2) software. Gene expression for both probes was normalized non-transgenic background. Fold changes were deemed significant at a p-value of ≤0.05, with statistical testing performed using the Student’s t-test.

## Results

Differentially expressed and alternatively spliced genes in both genotoxic and non-genotoxic compounds were assessed immediately after the end of treatment in a ATT-myc transgenic model.

### Tissue distribution of expression

The MouseExon10ST array contained 270,096 transcript clusters. After applying the aforementioned filters, 7,864 samples, each with between 4 and 200 probe sets, were analyzed. These samples were then evaluated for alternative splicing and differential gene expression using the previously discussed statistical methods. The number of genes (transcript clusters) expressed in each group for the tested transcript clusters is summarized as the following.

**Table:** 

Group	Number of transcript clusters with significant expression in each group
bht-1-mf	6,946—88.3% of genes tested
ndea-1-mf	7,198—91.5% of genes tested

Using the same test, the following table summarizes the frequencies of pair-wise co-expression between the study groups.

**Table:** 

	bht-1-mf	ndea-1-mf
bht-1-mf	6,946 (0,187)	6,759 (6,759)
ndea-1-mf	—	7,198 (0,439)

There were 6,946 genes where the bht-1-mf group showed significant expression above the background, while there were only 187 genes where this group was the only one with significant expression above the background. The numbers in parentheses indicate exclusivity, while the plain numbers represent the total count.

The following table summarizes all co-expression patterns. Since frequencies were exclusive, there were 6,759 genes that expressed the group bht-1-mf vs. ndea-1-mf and no other tissues.

**Table:** 

bht-1-mf vs. ndea-1-mf	6,759
bht-1-mf	187
ndea-1-mf	439

### Differential gene expression and alternative splicing

The statistical analysis detailed in the “*Methods*” section identified 2021 genes with significant exon–group interactions, indicating alternative splicing, as well as 645 genes with notable differences in gene expression between groups, including 181 genes with both expression differences and interactions. Tables 2, 3 present the top 10 genes showing significant differential alternative splicing and the top 10 genes with the highest fold changes in differential gene expression.

**TABLE 2:** Highlights the top 10 genes with significant fold changes in expression based on normalized, untransformed data.

	Gene symbol	TCluster ID	Description	Fold change	Differential Expression p-value
1	4432416J03Rik	6995384	RIKEN cDNA 4432416J03 gene	−4.11	7.27E-04
2	Tat	6979073	Tyrosine aminotransferase	1.81	2.15E-03
3	Fgfr1	6974743	Fibroblast growth factor receptor 1 (FGFR1)	2.01	1.40E-02
4	Atp8b1	6866118	ATPase class I type 8B member 1	−1.41	2.55E-02
5	Uqcr	6775372	Ubiquinol-cytochrome c reductase (6.4kD)	−1.71	2.91E-02
6	Grpel1	6929960	GrpE-like 1 mitochondrial	−1.41	2.88E-02
7	Las1l	7018304	LAS1-like (*S. cerevisiae*)	−1.31	2.47E-02
8	Slc38a2	6838257	Solute carrier family 38 member 2 (SLC)	2.31	2.32E-02
9	Nav1	6762429	Neuron navigator 1	1.31	2.16E-02
10	Pck1	6883654	Phosphoenolpyruvate carboxykinase 1 (PEPCK)	1.61	2.49E-02

**TABLE 3:** The top 10 genes with significant differential alternative splicing.

	Gene symbol	TCluster ID	Description	Exon–Tissue Interaction p-value
1	Dync1h1	6798108	Dynein cytoplasmic 1 heavy chain 1	1.66E-26
2	Acaca	6783063	Acetyl-coenzyme-A carboxylase alpha	3.49E-25
3	Acsm3	6963895	Acyl-CoAsynthetase medium-chain family	2.52E-17
4	Lrp1	6777957	Low-densitylipoprotein receptor-related	9.16E-17
5	Pzp	6957348	Pregnancy zone protein	1.49E-14
6	Hspg2	6917933	Perlecan (heparan sulfate proteoglycan 2)	1.64E-13
7	Abcb11	6887522	ATP-binding cassette sub-family B	4.70E-13
8	Tat	6979073	Tyrosine aminotransferase	6.45E-13
9	Tspan12	6952070	Tetraspanin 12	7.53E-13
10	Klkb1	6982094	Kallikrein B plasma 1	1.57E-12

Interestingly, as shown in Table 2, these genes are involved in several cellular processes and pathways that intersect in the context of cancer and genotoxicity.

### Comparison of differentially expressed genes and exons with known gene classifications

To detect significant overrepresentation in the GOMolFn, GOProcess, GOCellLoc, and Pathway categories, the 7,864 genes tested for differential alternative splicing and gene expression were compared with established gene classifications listed in the MouseExon10ST.info file. Contingency table analysis was used to identify groups where genes with significant splicing or expression differences were notably overrepresented. Under random circumstances, a group’s significant gene count had a hyper-geometric distribution (Ross, 2014), and the probability of seeing the given number or more significant genes in a group could be approximated as follows: the cumulative normal (x, mean = 0, variance = 1) was used, where x is equivalent to [a - (n *p)]/Sqr [n *p * (1-p)], the number of genes in the group is represented by n, the number of significant genes is represented by a, and the ratio of the total number of significant genes to the total number of genes is represented by p value.

### False discovery rate

The false discovery rate for this project was below 1.00E-00 for the tests of differential alternative splicing and gene expression based on calculations using the sequential step-down method described earlier.

This approach is like a one-sided Fisher exact test. The exact statistics can be calculated by summing all the discrete hypergeometric probability density function (PDF) values. Each group underwent this calculation, and the significant groups are listed below for each annotation class (i.e., where the function yields a result of less than 0.01 for either gene expression or alternative splicing).

### Significant representation in groups from the GOMolFn classification

Within the set of genes showing differential splicing or expression (as determined previously), 198 groups from the GOMolFn gene classification were significantly overrepresented. The depicted Table 3 lists the top 30 groups. Each row represents a group, with the three columns showing the group name, the number of tested genes with significant differential splicing (indicated by a p-value for overrepresentation), and the number of genes with significant differential gene expression (also indicated by a p-value for overrepresentation) (Table 4; Supplementary Figure S1).

**TABLE 4:** The top 30 groups of GOMolFn gene classifications that were significantly overrepresented in the set of differentially spliced or expressed genes.

	Number GE	Number AS	Group name
1	28 (8.70E-01)	161 (1.10E-09)	GO:0016491 oxidoreductase activity
2	0 (1.00-00)	14 (2.07E-07)	GO:0016627 oxidoreductase activity-Activity
3	19 (3.17E-01)	85 (3.39E-07)	GO:0016874 ligase activity
4	3 (5.49E-07)	1 (5.12E-01)	GO:0016847 1-aminocyclopropane-1-Carboxy
5	80 (1.35E-01)	284 (9.64E-07)	GO:0005524 ATP binding
6	2 (1.09E-06)	1 (2.15E-01)	GO:0005087 Ran guanyl-nucleotide Exchange
7	2 (1.09E-06)	1 (2.15E-01)	GO:0004311 farnesyltranstransferase activity
8	2 (1.09E-06)	0 (1.00-00)	GO:0005229 intracellular calcium Activation
9	4 (1.16E-06)	0 (1.00E-00)	GO:0042605 peptide antigen binding
10	1 (6.58E-01)	13 (3.05E-06)	GO:0003995 acyl-CoA dehydrogenase activity
11	5 (3.28E-06)	3 (4.51E-01)	GO:0000155 two-component sensor Activity
12	4 (8.06E-06)	3 (2.22E-01)	GO:0005520 insulin-like growth factor
13	96 (1.83E-01)	336 (1.12E-05)	GO:0000166 nucleotide binding
14	11 (2.38E-05)	14 (1.75E-01)	GO:0030170 pyridoxal phosphate Binding
15	4 (5.03E-01)	25 (2.41E-05)	GO:0050660 FAD binding
16	4 (8.57E-01)	36 (5.43E-05)	GO:0016887 ATPase activity
17	1 (8.14E-01)	16 (6.83E-05)	GO:0004177 aminopeptidase activity
18	0 (1.00E-00)	5 (7.07E-05)	GO:0y016717 oxidoreductase activity
19	5 (8.63E-05)	2 (8.35E-01)	GO:0016769 transferase activity-Transfer
20	4 (2.04E-02)	12 (9.12E-05)	GO:0015662 ATPase activity-coupled
21	3 (9.38E-05)	3 (8.61E-02)	GO:0005391sodium: potassium- exchanging A
22	36 (5.14E-02)	117 (9.51E-05)	GO:0003824 catalytic activity
23	26 (1.17E-01)	91 (1.04E-04)	GO:0008233 peptidase activity
24	2 (1.10E-04)	2 (5.20E-02)	GO:0017112 Rab guanyl-nucleotide Exchange
25	2 (1.10E-04)	2 (5.20E-02)	GO:0003989 acetyl-CoA carboxylase Activity
26	2 (1.10E-04)	2 (5.20E-02)	GO:0004075 biotin carboxylase Activity
27	2 (1.10E-04)	0 (1.00E-00)	GO:0004618 phosphoglycerate kinase Activity
29	2 (1.10E-04)	0 (1.00E-00)	GO:0042606 endogenous peptide antigen
30	3 (1.33E-01)	12 (2.19E-04)	GO:0016820 hydrolase activity-acting On acid anhydrides

### Significant representation in the groups from the GOProcess classification

Among the genes that showed differential splicing or expression (as previously described), 253 groups were significantly overrepresented in the GOProcess gene classification. The table below shows the top 30 groups. For each group, the table lists three columns: the number of tested genes with significant differential gene expression (indicated by a p-value for overrepresentation), the number of genes (418) with significant differential splicing (also indicated by a p-value for overrepresentation), and the group name (Table 5; Supplementary Figure 1).

**TABLE 5:** Shown top 30 Significant representation in the groups of the GOProcess classification.

	Number GE	Number AS	Group name
1	3 (3.33E-09)	0 (1.00E-00)	GO:0002504 antigen processing and cell express antigen
2	41 (7.55E-02)	149 (1.21E-07)	GO:0008152 metabolic process
3	0 (1.00E-00)	11 (5.67E-07)	GO:0000059 protein import into nucleus-
4	15 (9.08E-01)	99 (5.91E-07)	GO:0006118 electron transport
5	2 (8.45E-01)	27 (6.11E-07)	GO:0006631 fatty acid metabolic process
6	2 (1.09E-06)	0 (1.00E-00)	GO:0007527 adult somatic muscle
7	2 (1.09E-06)	0 (1.00E-00)	GO:0001659 thermoregulation
8	2 (1.09E-06)	0 (1.00E-00)	GO:0042755 eating behavior
9	2 (1.09E-06)	0 (1.00E-00)	GO:0030502 negative regulation of bone m
10	2 (1.09E-06)	0 (1.00E-00)	GO:0048002 antigen processing and presenting cell expresses peptide antigen
11	2 (1.09E-06)	0 (1.00E-00)	GO:0019883 antigen presentation, endogenous antigen
12	2 (1.09E-06)	0 (1.00E-00)	GO:0045582 positive regulation of T cell
13	2 (1.09E-06)	0 (1.00E-00)	GO:0002028 regulation of sodium ion transport
14	2 (1.09E-06)	0 (1.00E-00)	GO:0050873 brown fat cell differentiation
15	7 (6.13E-06)	3 (8.63E-01)	GO:0019882 antigen processing and presentation
16	3 (1.19E-05)	4 (2.0E-03)	GO:0018107 peptidyl-threonine phosphoryl
17	3 (1.19E-05)	2 (2.31E-01)	GO:0000050 urea cycle
18	1 (2.24E-01)	6 (1.53E-05)	GO:0009725 response to hormone stimulus
19	6 (2.29E-05)	4 (5.80E-01)	GO:0000160 two-component signal transducer
20	4 (3.64E-05)	2 (5.93E-01)	GO:0019886 antigen processing and presentation
21	1 (4.17E-01)	8 (4.17E-05)	GO:0006509 membrane protein ectodomain p
22	6 (5.00E-05)	7 (9.94E-02)	GO:0001503 ossification
23	2 (4.74E-03)	5 (7.0?E-05)	GO:0007044 cell-substrate junction assem
24	11 (3.16E-01)	48 (7.09E-05)	GO:0006629 lipid metabolic process
25	3 (9.38E-05)	4 (1.0?E-02)	GO:0019395 fatty acid oxidation
26	2 (1.10E-04)	0 (1.00E-00)	GO:0007028 cytoplasm organization and bi
27	2 (1.10E-04)	1 (3.80E-01)	GO:0006527 arginine catabolic process
28	2 (1.1OE-04)	0 (1.00E-00)	GO:0006415 translational termination
29	2 (1.1OE-04)	1 (3.80E-01)	GO:0006519 amino acid and derivative met
30	2 (1.1OE-04)	1 (3.80E-01)	GO:0030655 beta-lactam antibiotic metabolic process

### Significant representation in the groups from the GOCellLoc classification

Among the genes that exhibited differential splicing or expression (as outlined earlier), 41 groups were significantly overrepresented in the GOCellLoc gene classification. The table below presents the top 30 groups. Each row represents a single group, with the three columns indicating the number of tested genes with significant differential splicing (with a p-value for overrepresentation), the number of genes with significant differential gene expression (also with a p-value for overrepresentation), and the group name (Table 6; Supplementary Figure 1).

**TABLE 6:** Presents the top 30 GOCellLoc gene classifications that were significantly overrepresented in the set of differentially spliced or expressed genes.

	Number GE	Number AS	Group name
1	3 (3.33E-09)	0 (1.00E-00)	GO:0042405 nuclear inclusion body
2	3 (3.33E-09)	0 (1.00E-00)	GO:0042613 MHC class II protein complex
3	2 (1.09E-06)	1 (2.15E-01)	GO:0005577 fibrinogen complex
4	11 (3.49E-05)	10 (7.01E-01)	GO:0009897 external side of plasma membr
5	39 (6.56E-05)	72 (3.13E-01)	GO:0005886 plasma membrane
6	0 (1.00E-00)	5 (7.07E-05)	GO:0005579 membrane attack complex
7	2 (1.10E-04)	0 (1.00E-00)	GO:0016471 vacuolar portion- transporting
8	1 (2.78E-01)	6 (1.37E-04)	GO:0001740 Barr body
9	5 (1.92E-04)	5 (2.48E-01)	GO:0005741 mitochondrial outer membrane
10	1 (4.0?E-04)	1 (4.44E-02)	GO:0014069 postsynaptic density
11	1 (4.0?E-04)	1 (4.44E-02)	GO:0030055 cell-matrix junction
12	1 (4.0?E-04)	0 (1.00E-00)	GO:0045239 tricarboxylic acid cycle enzy
13	1 (4.0?E-04)	0 (1.00E-00)	GO:0005833 hemoglobin complex
14	1 (4.0?E-04)	0 (1.00E-00)	GO:0043235 receptor complex
15	1 (4.0?E-04)	0 (1.00E-00)	GO:0042101 T cell receptor complex
16	1 (4.0?E-04)	0 (1.00E-00)	GO:0016012 sarcoglycan complex
17	1 (4.0?E-04)	0 (1.00E-00)	GO:0042719 mitochondrial intermembrane s
18	1 (4.0?E-04)	0 (1.00E-00)	GO:0005964 phosphorylase kinase complex
19	1 (4.0?E-04)	1 (4.44E-02)	GO:0005796 Golgi lumen
20	1 (4.0?E-04)	1 (4.44E-02)	GO:0008280 cohesin complex
21	2 (4.12E-01)	12 (4.78E-04)	GO:0016459 myosin complex
22	29 (9 .30E-01)	148 (6.92E-04)	GO:0005783 endoplasmic reticulum
23	4 (7.92E-01)	30 (9.37E-04)	GO:0005777 peroxisome
24	2 (1.15E-03)	2 (1.33E-01)	GO:0005890 sodium:potassium- exchanging A
25	5 (1.22E-03)	2 (9.21 E-01)	GO:0005839 proteasome core complex (sens
26	61 (7.59E-01)	246 (1.25E-03)	GO:0005737 cytoplasm
27	6 (1.33E-03)	8 (1.95E-01)	GO:0016323 basolateral plasma membrane
28	0 (1.00E-00)	5 (2.78E-03)	GO:0046581 intercellular canaliculus
29	5 (5.99E-01)	27 (4 .00E-03)	GO:0005694 chromosome
30	18 (4.57E-03)	36 (1.SOE-01)	GO:0005794 Golgi apparatus

Connecting Genotoxicity and Cancer Pathways with Gene Ontology Terms Understanding how these molecular mechanisms affect cellular responses to DNA damage, cell survival, proliferation, and metastasis is crucial. This understanding is based on the Gene Ontology (GO-cellloc) terminology assigned to important pathways implicated in genotoxicity and carcinogenicity.

## Discussion

Genetic studies of cancer models play a crucial role in advancing our understanding of genotoxicity, cancer biology and developing new therapies. By studying the genetic mutations, alterations, and pathways involved in cancer, researchers can identify key biomarkers and therapeutic targets. These studies provide insights into the mechanisms of tumorigenesis, including how cells bypass normal growth control, evade the immune system, and metastasize.

Cancer models, particularly genetically engineered ones, allow for the examination of specific genetic changes in a controlled environment, helping to simulate the development genotoxicityand progression of cancer. This enables researchers to test potential therapies and assess their effectiveness in treating different types of cancer.

Furthermore, genetic research can aid in personalized medicine by identifying mutations that are specific to individual patients or cancer types, leading to more tailored and effective treatment strategies. Overall, genetic studies are essential for discovering novel therapeutic approaches, improving early detection, and advancing the development of targeted therapies that can more precisely address the genotoxicity and genetic underpinnings of cancer.

Classifying genes based on Gene Ontology, including molecular function, process, and cellular localization, is crucial for identifying key biomarkers and pathways that help distinguish between genotoxic and non-genotoxic carcinogens (Waters et al., 2010; Pérez et al., 2016).

Notably, these genes are linked through pathways pertaining to energy metabolism, cell signalling, stress responses, and genomic stability. These pathways are all altered in cancer and during genotoxic stress in the following ways, as shown in Table 2 based on Network or Pathway in Cancer and Genotoxicity: the functions of genes such as PEPCK, GrpE-like 1 mitochondrial, and ubiquinol-cytochrome c reductase in cellular energy metabolism which is changed in cancer—link them together. Mitochondrial failure can increase oxidative stress and DNA damage, contributing to genotoxicity and genomic instability, critical aspects in cancer formation (Sakai et al., 2012; Grasso et al., 2020).

The FGFR1 gene is essential for signalling pathways that control cell growth and survival, according to Growth and Survival Signalling. It may interact with metabolic pathways, and by encouraging unchecked cell division and resistance to genotoxic stress, its dysregulation may result in carcinogenesis (Yang et al., 2021).

Based on Cellular Stress and DNA Damage Response, Genes like LAS1-like, GrpE-like 1, and Tyrosine aminotransferase can be implicated in cellular stress responses, including protein folding and DNA repair. Their imbalance can contribute to genotoxic stress, increasing cancer cell survival despite DNA damage (Batra, 2013).

The SLC family and ATPase class I type 8B genes affect how cells process nutrients, ions, and medications, according to Transport and Drug Resistance. By altering the uptake of genotoxic drugs like chemotherapy, altered transport can affect the ability of cancer cells to survive in the presence of these agents (Alam et al., 2023).

Among the top ten genes with altered expression by the end of the DEN treatment when compared to BHT group is Phosphoenolpyruvate carboxykinase (PEPCK), which is involved in cellular energy metabolism an area that is significantly altered in cancer. PEPCK is traditionally recognized for its role in gluconeogenesis, but it also acts as a key regulator of the TCA cycle flux. This function of PEPCK connects metabolic flux and anabolic pathways to the proliferation of cancer cells (Montal et al., 2015).

Notably, the key metabolic pathways, particularly those involved in lipid synthesis, are altered in the setting of genotoxicity and carcinogenicity due to considerable differential alternative splicing. Specifically, genes like LRP, ACACA, and ACSM are linked to the altered metabolism of cancer cells, which promotes their proliferation, survival, and ability to withstand genotoxic stress. Below is a summary of their responsibilities: Acetyl-CoA Carboxylase Alpha, or ACACA, comes first: By changing acetyl-CoA into malonyl-CoA, an essential step in lipid synthesis, ACACA contributes to the production of fatty acids. An increase in fatty acid synthesis helps cancer cells maintain membrane integrity and prevent apoptosis, even in the face of DNA damage stress. Alternative splicing of ACACA can produce isoforms with altered enzymatic activity, which contribute to dysregulated lipid metabolism in cancer cells. This change in lipid synthesis supports the formation of cell membranes, which are essential for rapid cell division and survival under genotoxic stress and this expression recorded previously that the fatty acid metabolism is reprogrammed to promote the breast cancer progression (Zhao et al., 2021).

The medium-chain fatty acids are activated to their acyl-CoA derivatives by ACSM (Acyl-CoA Synthetase Medium Chain), which is necessary for cellular signalling and energy production (Yan et al., 2015). Alternative splicing of ACSM in cancer may result in the generation of isoforms with distinct cellular localisation or substrate preferences, thereby promoting altered lipid metabolism. This makes it easier for energy to be provided to promote rapid tumour growth and survival, allowing cancer cells to flourish in hypoxic or nutrient-deficient environments that are frequently linked to genotoxic stress (Shrestha et al., 2022).

The LRP (Low-Density Lipoprotein Receptor-Related Protein), which aids in the absorption of lipids, cholesterol, and other substances necessary for signalling and cellular structure maintenance. Different ligand affinities or changed internalisation properties can be produced by alternative splicing of LRP. By promoting lipid uptake and accumulation in cancer cells, these spliced isoforms may aid in the metabolic reprogramming of those cells. Higher lipid consumption promotes membrane biogenesis, prevents apoptosis, and helps cancer cells survive genotoxic stress by encouraging cell division (Fernández et al., 2020).

Cancer Cell Metabolic Shift Lipid synthesis and absorption are elevated in cancer cells as a result of the altered expression and splicing of these genes. This change facilitates a number of procedures. Additionally, energy production: In order to maintain their rapid development, cancer cells need a lot of energy. Because of the constant energy supply provided by altered lipid metabolism, cancer cells are able to avoid energy shortages that could otherwise result in cell death. Fifth, Membrane Formation: Because cancer cells have a higher capability for proliferating, they depend on the increased synthesis of lipids to create new cellular membranes and promote tumour growth. Lipid metabolism is essential for the formation of new membranes during cell division.

The Resistance to Genotoxic Stress, By preserving cellular integrity and function, the modified metabolic pathways aid cancer cells in fending off genotoxic stress. Despite the presence of genotoxic treatments like chemotherapy or radiation, cells with dysregulated lipid metabolism may be able to respond to DNA damage by avoiding apoptosis and repairing damaged DNA more efficiently, allowing for survival and development.

Through differential splicing, genes such as ACACA, ACSM, and LRP contribute to the altered metabolic pathways in cancer. This metabolic reprogramming allows cancer cells to avoid apoptosis, withstand genotoxic stress, and continue to proliferate, ultimately contributing to tumour progression and treatment resistance.

According to Growth and Survival Signalling, the FGFR1 gene, among the top ten genes with altered expression by the end of the DEN treatment when compared to BHT, which is highly expressed, is crucial for signalling pathways that regulate cell growth and survival. By promoting unrestrained cell division and resistance to genotoxic stress, it may interact with metabolic pathways. The FGFR1 gene has recently been found to be expressed in hepatocellular carcinoma, and the aberrant FGF/FGFR signalling in HCC initiation, progression, and therapy status offers fresh information on how to treat HCC (Wang et al., 2021).

By connecting these gene ontology terms to the molecular mechanisms of cancer and genotoxicity, we can observe the following:

In terms of Genomic Instability and DNA Damage Response, disruptions in DNA repair mechanisms (e.g., GO:0016887, GO:0016769) and oxidative stress (e.g., GO:0016491, GO:0016627) lead to the accumulation of mutations and chromosomal instability, both of which are key drivers of cancer development.

Under metabolic reprogramming, changes in cellular energy metabolism (e.g., GO:0016491, GO:0016627, GO:0016874) allow cancer cells to survive under stressful conditions, such as hypoxia and nutrient scarcity, which are frequently encountered in the tumor microenvironment. Thirdly, regarding Resistance to Genotoxic Agents, genes involved in cell signaling, drug resistance, and stress responses (e.g., GO:0005229, GO:0005087, GO:0004311, GO:0004618) help cancer cells resist therapies like chemotherapy and radiation by modulating survival and stress response pathways.

Concerning Tumor Growth and Metastasis, disruptions in growth and survival signaling pathways (e.g., GO:0005087, GO:0005524, GO:0017112) promote tumor progression and metastasis, allowing cancer cells to thrive under genotoxic stress and spread throughout the body. Collectively, the genes linked to these GO terms interact through intricate networks involving energy metabolism, stress responses, DNA repair, and drug resistance, all of which play essential roles in cancer development and response to genotoxic stress.

Genotoxic agents are known to induce neoplasm development, but many new pharmaceutical and environmental chemicals introduced into society should undergo testing to ensure their safety. Toxicogenomics involves applying genomic data to study the harmful effects of these chemicals with the aim of accelerating risk assessment and hazard screening processes (National Research Council, 2007).

Essential cellular functions involved in the cellular response to non-genotoxic and carcinogenicity stress are described by these gene ontology concepts. Cancer development, progression, and resistance to treatment are influenced by disruptions in DNA repair, cell cycle regulation, apoptosis resistance, immunological evasion, and cell migratory pathways (Li et al., 2007).

The following is an analysis based on the relevance of the Gene Ontology (GOPROCESS) terms you provided to genotoxicity and cancer, taking into account the pathways and molecular mechanisms they represent:

According to immune response and inflammation, GO:0002504 (Antigen Processing and Presentation of Peptide Antigen via MHC Class I): This process is involved in immune surveillance and the detection of abnormal cells, including cancer cells; disruption of this pathway can allow tumour cells to evade immune detection, facilitating tumour growth and metastasis (Dhatchinamoorthy et al., 2021). Furthermore, GO:0030502 (RNA Polymerase II Promoter-Mediated Transcription Regulation): Cancer frequently exhibits dysregulation of transcription factors or signalling pathways (e.g., NF-kB), which results in immunological suppression and genotoxic stress avoidance (Wang et al., 2004). Also, GO:0006118 (Oxidative Phosphorylation) is a crucial metabolic route involved in energy production, according to Energy Metabolism and Cellular Respiration. Oxidative phosphorylation is frequently changed in cancer cells to promote fast cell division (Solaini et al., 2011).

The accumulation of DNA damage and the advancement of cancer are significantly influenced by mitochondrial malfunction brought on by oxidative stress, which is frequently observed in genotoxicity. Additionally, GO:0000059 (Citrate Metabolic Process): Cancer cells commonly exhibit dysregulation of metabolic pathways such as the citric acid cycle. Tumour growth and survival are influenced by modifications in metabolic pathways, particularly in reaction to genotoxic stress (Icard et al., 2012).

According to Cell Cycle and Proliferation, GO:0007527 (Cell Differentiation), cancer is characterised by disrupted differentiation, which results in unchecked cell division (Williams and KJTJop, 2012). Tumour start and development are made possible by changes in differentiation processes, and genotoxic stress frequently makes these effects worse by producing DNA damage that encourages mutations in tumour suppressors and oncogenes. Additionally, GO:0001659 (Stem Cell Division Regulation): The division of stem cells is essential for both tumour development and tissue regeneration. Cancer stem cell populations that are resistant to genotoxic drugs like chemotherapy are a result of abnormal stem cell control (Patil et al., 2023).

Based on the DNA Repair and Genomic Stability, GO:0042755 (Telomere Maintenance): Many malignancies have telomere dysfunction. Shortening telomeres, which shield the ends of chromosomes, causes chromosomal instability, which is a key component in the development of cancer and the body’s reaction to genotoxic stress (Gilley et al., 2005). Additionally, the response to jasmonic acid (GO:0009725): In order to handle DNA damage brought on by genotoxic substances, this phrase refers to signalling pathways that might influence cell cycle regulation and DNA repair mechanisms (Christmann and Kaina, 2013).

Based on the GO:0048002 (Regulation of Cell Migration), which is based on Cell Migration and Invasion, cancer cells frequently show enhanced migration and invasion, both of which are necessary for metastasis. The ability of cancer cells to penetrate distant tissues is facilitated by genes that regulate migration; genotoxic stress can alter this process (Sinsong, 2016). Furthermore, the regulation of neurone differentiation (GO:0045582): Although this mechanism is mostly associated with neural differentiation, it may also interact with cancer metastasis pathways in nervous system tumours, where altered migration and differentiation lead to tumour invasion and resistance to genotoxic treatments (Desale et al., 2022).

Additionally, the GO:0002028 (Apoptotic Process) which is based on Apoptosis and Cellular Stress Responses: Evading apoptosis is a characteristic of cancer. Despite DNA damage from genotoxic chemicals, cancer cells can persist due to resistance to apoptosis. Cancer cells frequently exhibit inhibition of apoptotic pathways, which increases their resistance to genotoxic stress. Additionally, GO:0050873 (Cell Death Regulation): This route affects cancer cell resistance to chemotherapy by controlling the ratio of cell death to survival. The progression of tumours and chemoresistance are facilitated by disruption of cell death mechanisms. Furthermore, GO:0019883 (Regulation of Apoptotic Process): Signalling pathways that are closely linked to the regulation of apoptosis can be interfered with in cancer, resulting in resistance to genotoxic agents (Swift et al., 2014).

Notably, the GO:0018107 (Protein Acylation), which is based on Signal Transduction and Cancer Progression, acylation has an impact on how well proteins function in signal transduction pathways that control cell growth and survival. Protein acylation changes in cancer can encourage aberrant signalling, which aids in tumour growth and genotoxic agent resistance. Additionally, GO:0000160 (Biosynthesis of Inositol Phosphate): The proliferation, survival, and resistance to genotoxic stress of cancer cells are frequently linked to the dysregulation of inositol phosphates, which are essential for cellular signalling (Chakraborty, 2018).

The tumour suppression and protein metabolism are crucial for controlling the cell cycle and apoptosis (GO:0006509 (Protease Activity). Proteases in cancer may promote metastasis and treatment resistance by facilitating tumour cell motility, invasion, and the degradation of extracellular matrix components (Rakashanda et al., 2012). Additionally. GO:0001503 (Biosynthesis of Chondroitin Sulphate): The extracellular matrix contains chondroitin sulphate. Resistance to genotoxic stress and cancer metastasis are linked to changes in the production of matrix components. Additionally, the Cell-Cycle Phase Transition (GO:0007044): Genomic stability depends on the appropriate control of cell-cycle progression. One important characteristic of cancer is unchecked growth, which is frequently caused by changes in cell-cycle regulation.

Based on the GOPROCESS, the uptake and efflux of chemotherapeutic medications and other genotoxic chemicals might be impacted by altered transport pathways, according to cellular communication and tumour microenvironment (Vaidya et al., 2022), GO:0006629 (Regulation of Transport). The ability of cancer cells to endure genotoxic stress can be significantly influenced by the modulation of transport. Furthermore, GO:0007028 (Organelle Fission): Cell survival and mitochondrial function are regulated by mitochondrial fission. The survival and resilience of cancer cells to genotoxic stress are influenced by altered mitochondrial dynamics. Additionally, GO:0019395 (Pyrimidine Deoxyribonucleotide Biosynthesis Process): Uncontrolled cell proliferation in cancer may be a result of nucleotide biosynthesis dysregulation. Deoxyribonucleotide buildup can also impact DNA repair systems, increasing the number of mutations in cancer cells (Robinson et al., 2020).

Based on the GOPROCESS key mechanisms, the molecular mechanisms are essential to understand the genotoxicity reactions and the genesis of cancer. Cancer cells survive and multiply under genotoxic stress through a variety of basic processes, including metabolic reprogramming, tumour invasion, resistance to apoptosis, altered cell signalling, and genomic instability. A favourable environment for cancer progression and therapeutic resistance is produced by dysregulation in various pathways, whether they be in energy metabolism, apoptosis, DNA repair, or drug resistance. Cancer’s complicated biochemistry and how it reacts to genotoxic treatments like radiation or chemotherapy are caused by the intersection of several of these mechanisms (Sasaki et al., 2020).

To enhance and expedite chemical testing, we propose the use of the ATT-myc transgenic model and exon arrays to identify gene expression differences that aid in chemical categorization. We specifically selected the liver as the target organ due to its role in biotransforming a variety of compounds and its ability to activate the toxicity of substances through the induction of cytochrome P450 enzymes (Ioannides and Lewis, 2004), which increase the electrophilicity of pro-carcinogens. We used exon arrays to conduct gene expression profiling and applied Biotique analysis systems and statistical methods for data analysis.

The analysis was conducted on 13 November 2010, using XRAY (version 3.2) software, an Excel add-in from Biotique Systems Inc. (Burke, 2007). This document was automatically generated by XRAY. The 11 input CEL files were analyzed with the Affymetrix MouseExon10ST array to identify genes that were significantly differentially expressed or showed other notable genes with significant differential alternative splicing between the groups of interest (Gardina et al., 2006; Huang et al., 2007; Clark et al., 2007).

Using mixed-model analysis of variance, we examined six hybridizations that were conducted immediately after the treatment with both genotoxic diethylnitrosamine and non-genotoxic butylated hydroxytoluene on a MouseExon10ST array. Out of 645 genes with significant expression differences between the groups and 2021 genes showing significant exon–group interactions (indicative of alternative splicing), 181 genes exhibited both gene and potential splicing differences (p < 0.01). Among the most significant genes, TAT encodes the mitochondrial enzyme tyrosine aminotransferase, which is predominantly found in the liver and metabolizes tyrosine into toxic molecules that are either excreted by the kidneys or utilized in energy-producing reactions (Shiman and Gray, 1998). Furthermore, TAT is a novel tumor suppressor gene (TSG), and its inactivation due to gene deletion and hypermethylation plays a role in the development of hepatocellular carcinoma (HCC) (Fu et al., 2010; Mehere et al., 2010). Additionally, HNF-4, a transcription factor whose expression is reduced in the albino-lethal liver, is crucial for the expression of TAT (Kelsey et al., 1992).

Among the most significant genes, fibroblast growth factors (FGF-1 and FGF-2) are heparin-binding factors that promote the proliferation, migration, and differentiation of neuroectodermal and mesodermal cells. These fibroblast growth factors are also widely expressed in adult tissues, particularly at sites of injury (Gospodarowicz et al., 1987; Burgess and Maciag, 1989; Basilico and Moscatelli, 1992; Mason, 1994).

In the GOMolFn gene classification, 198 groups were significantly overrepresented among the differentially spliced or expressed genes. Previous research suggests that a mechanistic approach is a promising strategy for both prediction and functional category analysis or pathway identification. Notably, the most significant genes were related to oxidative stress, lipid metabolism, and genes associated with pregnancy. This finding aligns with the known effects of genotoxins and the role of oxidative stress in carcinogenesis (Hernández et al., 2009; Gurer-Orhan et al., 2006). Additionally, the presence of genes related to lipid metabolism is typical of profiles delivered by peroxisome proliferators.

The alternative splicing of pregnancy-related genes might be linked to fetal malformations associated with genotoxic chemicals. Alternative splicing is crucial in various regulatory processes and diseases. Identifying genetic variants that influence splicing phenotypes is essential for understanding how genetic variations impact alternative splicing (Yang et al., 2017).

The relationship between these GO keywords and important genotoxicity and cancer pathways is examined below: According to DNA Damage and Repair, first Phospholipids have a role in preserving the integrity of cellular membranes (GO:0042405; Phospholipid Binding) (Benedict, 2020). Lipid metabolism may change in response to genotoxic stress, impacting cellular structural stability and repair processes. Mitochondrial Outer Membrane GO:0005741: One of the main causes of apoptosis is mitochondrial malfunction brought on by genotoxic stress. Damage to the mitochondria may cause cancer cells to become resistant to apoptosis, allowing them to survive DNA damage. Additionally, Nucleotide binding (GO:0005964): This process is involved in DNA replication and repair. A major characteristic of carcinogenesis, chromosomal instability and mutations are more likely when nucleotide metabolism is dysregulated because it can disrupt DNA repair pathways. GO:0042719 (Cholesterol Metabolic Process): Cellular signalling, including pathways that control DNA repair and death, and membrane composition are all impacted by dysregulated cholesterol metabolism. Changes in the metabolism of cholesterol are associated with the advancement of cancer (Mehere et al., 2010).

According to cell cycle regulation, signals that regulate the advancement of the cell cycle are mediated by receptors on the plasma membrane GO:0009897 (Negative Regulation of Receptor Activity) (Li et al., 2016). Also, Uncontrolled proliferation in response to genotoxic stress can result from the loss of receptor activation, which is frequently observed in cancer cells. GO:0005783 (Endoplasmic Reticulum Membrane): The ER plays a role in the folding and synthesis of proteins, both of which are critical during reactions to DNA damage. The unfolded protein response (UPR), which can be triggered by genotoxic stress, can accelerate the development of cancer if it is dysregulated. GO:0008280 (Regulation of Cell Proliferation): One of the hallmarks of cancer is unchecked cell proliferation. When under genotoxic stress, this process is frequently dysregulated, which results in the development and spread of tumours. Synaptic Vesicle GO:0005890: Although the primary function of synaptic vesicles is neurotransmission, their involvement in cellular signalling may have an impact on the proliferation and stress response of cancer cells (Makrygianni and Chrousos, 2023).

Considering the growth and metastasis of tumours, one of the main characteristics of cancer metastasis is increased migration (GO:0016471; Cell Migration). The spread of cancer cells to distant organs can be facilitated by genotoxic stress, which can change cell migratory routes. Additionally, GO:0001740 (Retinal Pigment Epithelium Development): Although particular to the eye, this phrase implies that genotoxic stress influences cell fate and tumour dissemination, and that altered differentiation and migration may be involved in eye malignancies. Moreover, GO:0046581 (Regulation of Epithelial Cell Migration): Genotoxic stress can enhance migration, which aids in the spread of cancer cells. Epithelial cell migration is implicated in cancer metastasis (Bharadwaj and Mandal, 2020).

Based on homeostasis and cellular communication, Cytoplasmic Membrane-Bounded Vesicle (GO:0005579): These vesicles take part in transport and signalling within cells (Samanta et al., 2024). Dysregulation of vesicular transport can impair stress responses and contribute to cancer cell survival under genotoxic conditions. Additionally, GO:0005694 (Chromosome): One of the hallmarks of cancer is chromosomal instability, which is frequently brought on by genotoxic chemicals. Mutations brought on by genomic instability cause oncogenes to become active and tumour suppressor genes to become silenced. Moreover, GO:0005794 (Golgi Apparatus): The Golgi apparatus is involved in protein modification and sorting. Its malfunction can change cellular signalling and encourage the formation of tumours, especially when it occurs under genotoxic stress (Bui et al., 2021).

Inconclusions, the essential cellular functions involved in the cellular response to carcinogenicity and genotoxic stress are described by these gene ontology concepts. Cancer development, progression, and resistance to treatment are influenced by disruptions in DNA repair, cell cycle regulation, apoptosis resistance, immunological evasion, and cell migratory pathways. These changed pathways in cancer cells mitigate the DNA damage and oxidative stress that genotoxic treatments, such radiation or chemotherapy, usually cause. Finding therapeutic targets and methods for overcoming cancer treatment resistance are made easier with an understanding of these important biological pathways. Finally, the bioinformatic of exon array analysis identify the liver tumor genetics in ATT-myc mouse model of liver cancer and enable distinguished molecular pathway of both genotoxic and non-genotoxic carcinogens. Classifying genes based on Gene Ontology, including molecular function, process, and cellular localization, is crucial for identifying key biomarkers and pathways that help for future development of therapeutics agents.

## Data Availability

The datasets presented in this study can be found in online repositories. The names of the repository/repositories and accession number(s) can be found in the article/Supplementary Material.

## References

[B1] Alam S. Doherty E. Ortega-Prieto P. Arizanova J. Fets L. J. D. M. (2023). Membrane transporters in cell physiology, cancer metabolism and drug response. Mechanisms 16 (11), dmm050404. 10.1242/dmm.050404 PMC10695176 38037877

[B2] Ames B. N. Lee F. D. Durston W. E. (1973). An improved bacterial test system for the detection and classification of mutagens and carcinogens. Proc. Natl. Acad. Sci. U S A 70 (3), 782–786. 4577135 10.1073/pnas.70.3.782 PMC433358

[B3] Basilico C. Moscatelli D. J. A. (1992). The FGF family of growth factors and oncogenes. Adv. Cancer Res. 59, 115–165. 10.1016/s0065-230x(08)60305-x 1381547

[B4] Batra R. (2013). Computational methods to analyze image-based siRNA knockdown screens. Computer Science, Medicine, Biology. 10.11588/HEIDOK.00014922

[B5] Benedict T. J. (2020). Molecular organization of mammalian epithelial tight junctions. Singapore: National University of Singapore.

[B6] Benjamini Y. Hochberg Y. (1995). Controlling the false discovery rate: a practical and powerful approach to multiple testing. J. Royal Statis. Soc. Series B Methodol. 57 (1), 289–300.

[B7] Bharadwaj D. Mandal M. J. C. (2020). Senescence in polyploid giant cancer cells: a road that leads to chemoresistance. Cytokine. Growth Factor Reviews 52, 68–75. 31780423 10.1016/j.cytogfr.2019.11.002

[B75] Bolstad B. M. Irizarry R. A. Astrand M. Speed T. P. (2003). A comparison of normalization methods for high density oligonucleotide array data based on variance and bias. Bioinformatics. 19 (2), 185–193. 10.1093/bioinformatics/19.2.185 12538238

[B8] Bui S. Mejia I. Díaz B. Wang Y. J. F. (2021). Adaptation of the Golgi apparatus in cancer cell invasion and metastasis. Front. Cell Dev. Biol. 9, 806482. 10.3389/fcell.2021.806482 34957124 PMC8703019

[B9] Burgess W. H. Maciag T. (1989). The heparin-binding (fibroblast) growth factor family of proteins. Annu. Rev. Biochem. 58 (1), 575–602. 2549857 10.1146/annurev.bi.58.070189.003043

[B10] Chakraborty A (2018). The inositol pyrophosphate pathway in health and diseases. Biol. Rev. Camb. Philos. Soc. 93 (2), 1203–1227. 29282838 10.1111/brv.12392 PMC6383672

[B11] Chen L. Huang W. Wang L. Zhang Z. Zhang F. Zheng S. (2020). The effects of epigenetic modification on the occurrence and progression of liver diseases and the involved mechanism. Expert Rev. Gastroenterol. Hepatol. 14 (4), 259–270. 10.1080/17474124.2020.1736042 32124651

[B12] Choy W. Assessment C. R. (2001). Genotoxic and non-genotoxic mechanisms of carcinogenesis, in book: genetic toxicology and cancer risk assessment 47–72.

[B13] Christmann M. Kaina B. (2013). Transcriptional regulation of human DNA repair genes following genotoxic stress: trigger mechanisms, inducible responses and genotoxic adaptation. Nucleic. Acids. Res. 41 (18), 8403–8420. 23892398 10.1093/nar/gkt635 PMC3794595

[B14] Clark T. A. Schweitzer A. C. Chen T. X. Staples M. K. Lu G. Wang H. (2007). Discovery of tissue-specific exons using comprehensive human exon microarrays. Genome Biol. 8 (4), R64–R16. 10.1186/gb-2007-8-4-r64 17456239 PMC1896007

[B16] Dalemans W. Perraud F. Le Meur M. Gerlinger P. Courthey M. Pavirani A. J. B. (1990). Heterologous protein expression by transimmortalized differentiated liver cell lines derived from transgenic mice (hepatomas/alpha 1 antitrypsin/ONC mouse). Biologicals 18 (3), 191–198. 10.1016/1045-1056(90)90006-l 2257132

[B17] Desale H. Buekens P. Alger J. Cafferata M. L. Harville EW Herrera C. (2022). Epigenetic signature of exposure to maternal Trypanosoma cruzi infection in cord blood cells from uninfected newborns. Epigenomics. 14 (15), 913–927. 10.2217/epi-2022-0153 36039408 PMC9475499

[B18] Desaulniers D. Vasseur P. Jacobs A. Aguila M. C. Ertych N. Jacobs M. (2021). Integration of epigenetic mechanisms into non-genotoxic carcinogenicity hazard assessment: focus on DNA methylation and histone modifications. Int. J. Mol. Sci. 22 (20), 10969. 10.3390/ijms222010969 34681626 PMC8535778

[B19] Dhatchinamoorthy K. Colbert J. D. Kenneth L. R. (2021). Cancer immune evasion through loss of MHC class I antigen presentation. Front. Immunol. 12, 636568. 10.3389/fimmu.2021.636568 33767702 PMC7986854

[B20] Ellinger-Ziegelbauer H. Stuart B. Wahle B. Bomann W. Ahr H.-J. (2004). Characteristic expression profiles induced by genotoxic carcinogens in rat liver. Toxicol. Sci. 77 (1), 19–34. 10.1093/toxsci/kfh016 14600272

[B21] Fernández L. P. Gomez de Cedron M. Ajfio R. de M. (2020). Alterations of lipid metabolism in cancer: implications in prognosis and treatment. Front. Oncol. 10, 577420. 10.3389/fonc.2020.577420 33194695 PMC7655926

[B22] Fu L. Dong S. S. Xie Y. W. Tai L. S. Chen L. Kong K. L. (2010). Down‐regulation of tyrosine aminotransferase at a frequently deleted region 16q22 contributes to the pathogenesis of hepatocellular carcinoma. Hepatology 51 (5), 1624–1634. 10.1002/hep.23540 20209601

[B23] Furihata C. Watanabe T. Suzuki T. Hamada S. Nakajima M. J. G. Environment (2016). Collaborative studies in toxicogenomics in rodent liver in JEMS MMS; a useful application of principal component analysis on toxicogenomics. Genes Environ. 38 (1), 15–10. 10.1186/s41021-016-0041-0 27482301 PMC4968012

[B24] Gardina P. J. Clark T. A. Shimada B. Staples M. K. Yang Q. Veitch J. (2006). Alternative splicing and differential gene expression in colon cancer detected by a whole genome exon array. BMC Genomics 7, 325–418. 10.1186/1471-2164-7-325 17192196 PMC1769375

[B25] Gilley D. Tanaka H. Herbert B.-S. (2005). Telomere dysfunction in aging and cancer. Cancer 37 (5), 1000–1013. 10.1016/j.biocel.2004.09.003 15743674

[B26] Gospodarowicz D. Neufeld G. Schweigerer L. J. (1987). Fibroblast growth factor: structural and biological properties. J. Cell. Physiol. Suppl. 133 (S5), 15–26. 10.1002/jcp.1041330405 2824530

[B27] Grasso D. Zampieri L. X. Capelôa T. Van de Velde J. A. Sonveaux P. J. C. (2020). Mitochondria in cancer. Cell Stress 4 (6), 114–146. 10.15698/cst2020.06.221 32548570 PMC7278520

[B28] Gurer-Orhan H. Orhan H. Vermeulen N. P. Meerman J. Screening H. T. (2006). Screening the oxidative potential of several mono-and di-halogenated biphenyls and biphenyl ethers in rat hepatocytes. Comb. Chem. High. Throughput Screen. 9 (6), 449–454. 10.2174/138620706777698517 16842226

[B29] Hartwig A. Arand M. Epe B. Guth S. Jahnke G. Lampen A. (2020). Mode of action-based risk assessment of genotoxic carcinogens. Arch. Toxicol. 94, 1787–1877. 10.1007/s00204-020-02733-2 32542409 PMC7303094

[B30] Hernández L. G. van Steeg H. Luijten M. JJMRRiMR van B. (2009). Mechanisms of non-genotoxic carcinogens and importance of a weight of evidence approach. Mutat. Res. 682 (2-3), 94–109. 10.1016/j.mrrev.2009.07.002 19631282

[B31] Huang R. S. Duan S. Bleibel W. K. Kistner E. O. Zhang W. Clark T. A. (2007). A genome-wide approach to identify genetic variants that contribute to etoposide-induced cytotoxicity. Proc. Natl. Acad. Sci. U. S. A. 104 (23), 9758–9763. 10.1073/pnas.0703736104 17537913 PMC1887589

[B32] Hueper K. Elalfy M. Laenger F. Halter R. Rodt T. Galanski M. (2012). PET/CT imaging of c-Myc transgenic mice identifies the genotoxic N-nitroso-diethylamine as carcinogen in a short-term cancer bioassay. PLoS One 7 (2), e30432. 10.1371/journal.pone.0030432 22319569 PMC3271108

[B33] Icard P. Poulain L. Hubert L. (2012). Understanding the central role of citrate in the metabolism of cancer cells. Biochimica et Biophysica Acta (BBA) 1825 (1), 111–116. 22101401 10.1016/j.bbcan.2011.10.007

[B34] Ioannides C. Lewis D. F. V. (2004). Cytochromes P450 in the bioactivation of chemicals. Curr. Top. Med. Chem. 4 (16), 1767–1788. 15579107 10.2174/1568026043387188

[B35] Jacobs M. N. Colacci A. Louekari K. Luijten M. Hakkert B. C. Paparella M. (2016). International regulatory needs for development of an IATA for non-genotoxic carcinogenic chemical substances. ALTEX 33 (4), 359–392. 10.14573/altex.1601201 27120445

[B36] Kelsey G. Ruppert S. Schedl A. Schmid E. Thies E. Schütz G. (1992). Multiple effects on liver-specific gene expression in albino lethal mice caused by deficiency of an enzyme in tyrosine metabolism. J. Cell Sci. Suppl. 1992 (Suppl. ment_16), 117–122. 10.1242/jcs.1992.supplement_16.14 1297646

[B37] Lee S. J. Yum Y. N. Kim S. C. Kim Y. Lim J. Lee W. J. (2013). Distinguishing between genotoxic and non-genotoxic hepatocarcinogens by gene expression profiling and bioinformatic pathway analysis. Sci. Rep. 3 (1), 2783. 10.1038/srep02783 24089152 PMC6505678

[B38] Lee W. J. Kim S. C. Lee S. J. Lee J. Park J. H. Yu K.-S. (2014). Investigating the different mechanisms of genotoxic and non-genotoxic carcinogens by a gene set analysis. PLoS One 9 (1), e86700. 10.1371/journal.pone.0086700 24497971 PMC3908933

[B39] Li H.-H. Aubrecht J. Fornace Jr A. Mmo M. (2007). Toxicogenomics: overview and potential applications for the study of non-covalent DNA interacting chemicals. Mutat. Res. 623 (1-2), 98–108. 10.1016/j.mrfmmm.2007.03.013 17548094

[B40] Li J. Huang Z. Wei L. (2016). Bioinformatics analysis of the gene expression profile of hepatocellular carcinoma: preliminary results. Contemp. Oncol. 20 (1), 20–27. 10.5114/wo.2016.58497 PMC4829745 27095935

[B41] Makrygianni E. A. Chrousos GPJN (2023). Extracellular vesicles and the stress system. Neuroendocrinology 113 (2), 120–167. 10.1159/000527182 36137504

[B42] Mason I. (1994). The ins and outs of fibroblast growth factors. Cell 78, 547–552. 10.1016/0092-8674(94)90520-7 8069907

[B43] Mehere P. Han Q. Lemkul J. A. Vavricka C. J. Robinson H. Bevan D. R. (2010). Tyrosine aminotransferase: biochemical and structural properties and molecular dynamics simulations. Protein Cell 1 (11), 1023–1032. 10.1007/s13238-010-0128-5 21153519 PMC3023147

[B44] Mjcl D. (2012). Oxidatively induced DNA damage: mechanisms, repair and disease. Cancer Lett. 327 (1-2), 26–47. 10.1016/j.canlet.2012.01.016 22293091

[B45] Montal E. D. Dewi R. Bhalla K. Ou L. Hwang B. J. Ropell A. E. (2015). PEPCK coordinates the regulation of central carbon metabolism to promote cancer cell growth. Mol. Cell 60 (4), 571–583. 10.1016/j.molcel.2015.09.025 26481663 PMC4656111

[B46] Montgomery D. C. (2017). Design and analysis of experiments. John Wiley and Sons.

[B15] National Research Council (2007). Applications of Toxicogenomic Technologies to Predictive Toxicology and Risk Assessment. Washington, DC: The National Academies Press. 10.17226/12037 20669432

[B47] Patil T. Rohiwal S. S. Tiwari A. P. Therapy (2023). Stem cells: therapeutic implications in chemotherapy and radiotherapy resistance in cancer therapy. Curr. Stem Cell Res. Ther. 18 (6), 750–765. 10.2174/1574888X17666221003125208 36200212

[B48] Pérez L. O. González-José R. García P. P. (2016). Prediction of non-genotoxic carcinogenicity based on genetic profiles of short term exposure assays. Toxicol. Res. 32, 289–300. 10.5487/tr.2016.32.4.289 27818731 PMC5080858

[B49] Rahmanian N. Shokrzadeh M. Eskandani M. J. (2021). Recent advances in γH2AX biomarker-based genotoxicity assays: a marker of DNA damage and repair. DNA Repair 108, 103243. 10.1016/j.dnarep.2021.103243 34710661

[B50] Rajan P. K. Udoh U.-A. Sanabria J. D. Banerjee M. Smith G. Schade M. S. (2020). The role of histone acetylation-/methylation-mediated apoptotic gene regulation in hepatocellular carcinoma. Int. J. Mol. Sci. 21 (23), 8894. 10.3390/ijms21238894 33255318 PMC7727670

[B51] Rakashanda S. Rana F. Rafiq S. Masood A. Amin S. (2012). Role of proteases in cancer. Biotechnol. Mol. Biol. 7 (4), 90–101. 10.5897/BMBR11.027

[B52] Robinson A. D. Eich M.-L. Sjcl V. (2020). Dysregulation of *de novo* nucleotide biosynthetic pathway enzymes in cancer and targeting opportunities. 470:134–140. 10.1016/j.canlet.2019.11.013 31733288

[B53] Ross S. M. (2014). Introduction to probability models. Academic Press.

[B54] Sakai C. Tomitsuka E. Esumi H. Harada S. Kita K. (2012). Mitochondrial fumarate reductase as a target of chemotherapy: from parasites to cancer cells. Biochim. Biophys. Acta 1820 (5), 643–651. 10.1016/j.bbagen.2011.12.013 22226661

[B55] Samanta A. Yoo M.-J. Koh J. Lufkin T. Kraus P. (2024). Small extracellular vesicles by nucleus pulposus cells maintain niche and cell homeostasis via receptor shuffling and metabolic enzyme supplements. bioRxiv preprint. 10.1101/2024.12.12.628054

[B56] Santoni-Rugiu E. Preisegger K. H. Kiss A. Audolfsson T. Shiota G. Schmidt E. V. (1996). Inhibition of neoplastic development in the liver by hepatocyte growth factor in a transgenic mouse model. Proc. Natl. Acad. Sci. U. S. A. 93 (18), 9577–9582. 10.1073/pnas.93.18.9577 8790372 PMC38470

[B57] Sasaki J. C. Allemang A. Bryce S. M. Custer L. Dearfield K. L. Dietz Y. (2020). Application of the adverse outcome pathway framework to genotoxic modes of action. Environ. Mol. Mutagen. 61 (1), 114–134. 10.1002/em.22339 31603995

[B58] Shiman R. Gray D. W. (1998). Formation and fate of tyrosine: intracellular partitioning of newly synthesized tyrosine in mammalian liver. J. Biol. Chem. 273 (52), 34760–34769. 10.1074/jbc.273.52.34760 9857000

[B59] Shrestha R. Nassar Z. D. Hanson A. R. Iggo R. Townley S. L. Dehairs J. (2022). ACSM1 and ACSM3 regulate prostate cancer fatty acid metabolism to promote tumour growth and constrain ferroptosis. 10. 13.511039. 10.1158/0008-5472.CAN-23-1489 38657108

[B60] Simes R. J. J. B (1986). An improved Bonferroni procedure for multiple tests of significance. Biometrika 73 (3), 751–754. 10.2307/2336545

[B61] Sinsong (2016). Gene expression profiling of breast cancer according to mammographic microcalcifications: graduate school of seoul national university.

[B62] Solaini G. Sgarbi G. Baracca A. (2011). Oxidative phosphorylation in cancer cells. Biochim. Biophys. Acta 1807 (6), 534–542. 10.1016/j.bbabio.2010.09.003 20849810

[B63] Swift L. H. Rmjijoms G. (2014). Genotoxic anti-cancer agents and their relationship to DNA damage, mitosis, and checkpoint adaptation in proliferating cancer cells. Int J Mol Sci 15 (3), 3403–3431. 24573252 10.3390/ijms15033403 PMC3975345

[B64] Vaidya F. U. Sufiyan C. A. Mishra V. Gupta V. K. Rawat S. G. Kumar A. (2022). Molecular and cellular paradigms of multidrug resistance in cancer. Cancer Rep. Hob. 5 (12), e1291. 10.1002/cnr2.1291 PMC9780431 33052041

[B65] Van Delft J. Van Agen E. Van Breda S. Herwijnen M. Staal Y. Kleinjans J. J. C. (2004). Discrimination of genotoxic from non-genotoxic carcinogens by gene expression profiling. Carcinogenesis 25 (7), 1265–1276. 10.1093/carcin/bgh108 14963013

[B66] Wang Y. Liu D. Zhang T. Xia L. J. C. (2021). FGF/FGFR signaling in hepatocellular carcinoma: from carcinogenesis to recent therapeutic intervention. Cancers (Basel). 13 (6), 1360. 10.3390/cancers13061360 33802841 PMC8002748

[B67] Wang Y. Seimiya M. Kawamura K. Yu L. Ogi T. Takenaga K. (2004). Elevated expression of DNA polymerase κ in human lung cancer is associated with p53 inactivation: negative regulation of POLK promoter activity by p53. Int. J. Oncol. 25 (1), 161–165. 10.3892/ijo.25.1.161 15202001

[B68] Waters M. D. Jackson M. Lea I. (2010). Characterizing and predicting carcinogenicity and mode of action using conventional and toxicogenomics methods. Mutat. Res. 705 (3), 184–200. 10.1016/j.mrrev.2010.04.005 20399889

[B69] Williams G. H. KJTJop S. (2012). cell cycle cancer 226 (2), 352–364. 10.1002/path.3022 21990031

[B70] Yan S. Yang X.-F. Liu H.-L. Fu N. Ouyang Y. (2015). Long-chain acyl-CoA synthetase in fatty acid metabolism involved in liver and other diseases: an update. World J. Gastroenterol. 21 (12), 3492–3498. 10.3748/wjg.v21.i12.3492 25834313 PMC4375570

[B71] Yang Q. Hu Y. Li J. Zhang X. J. B. (2017). ulfasQTL: an ultra-fast method of composite splicing QTL analysis. BMC Genomics 18 (1), 963–969. 10.1186/s12864-016-3258-1 28198669 PMC5310271

[B72] Yang Z. Liang S. Q. Saliakoura M. Yang H. Vassella E. Konstantinidou G. (2021). Synergistic effects of FGFR1 and PLK1 inhibitors target a metabolic liability in KRAS‐mutant cancer. EMBO Mol. Med. 13 (9), e13193. 10.15252/emmm.202013193 34369083 PMC8422071

[B73] Zhao J. Xie F. Yang Y. Wang S. (2021). Reprogramming of fatty acid metabolism in breast cancer: a narrative review. Transl. Breast Cancer Res. 2 (1), 5. 10.21037/tbcr-20-53

[B74] Zhao Y. Xie P. Fan H. (2012). Genomic profiling of microRNAs and proteomics reveals an early molecular alteration associated with tumorigenesis induced by MC-LR in mice. Environ. Sci. Technol. 46 (1), 34–41. 10.1021/es201514h 21882851

