# Supplementary material for: Differentially expressed and alternately spliced genes as a novel tool for genotoxicity: a computerized study in ATT-myc transgenic mice for the recognition of genotoxic and non-genotoxic chemical
Source: Front Genet. 2025 Mar 28;16:1505379. doi: 10.3389/fgene.2025.1505379 (PMC11986717; doi:10.3389/fgene.2025.1505379)
Supplement: Supplementary file 1 [file Image1.pdf]

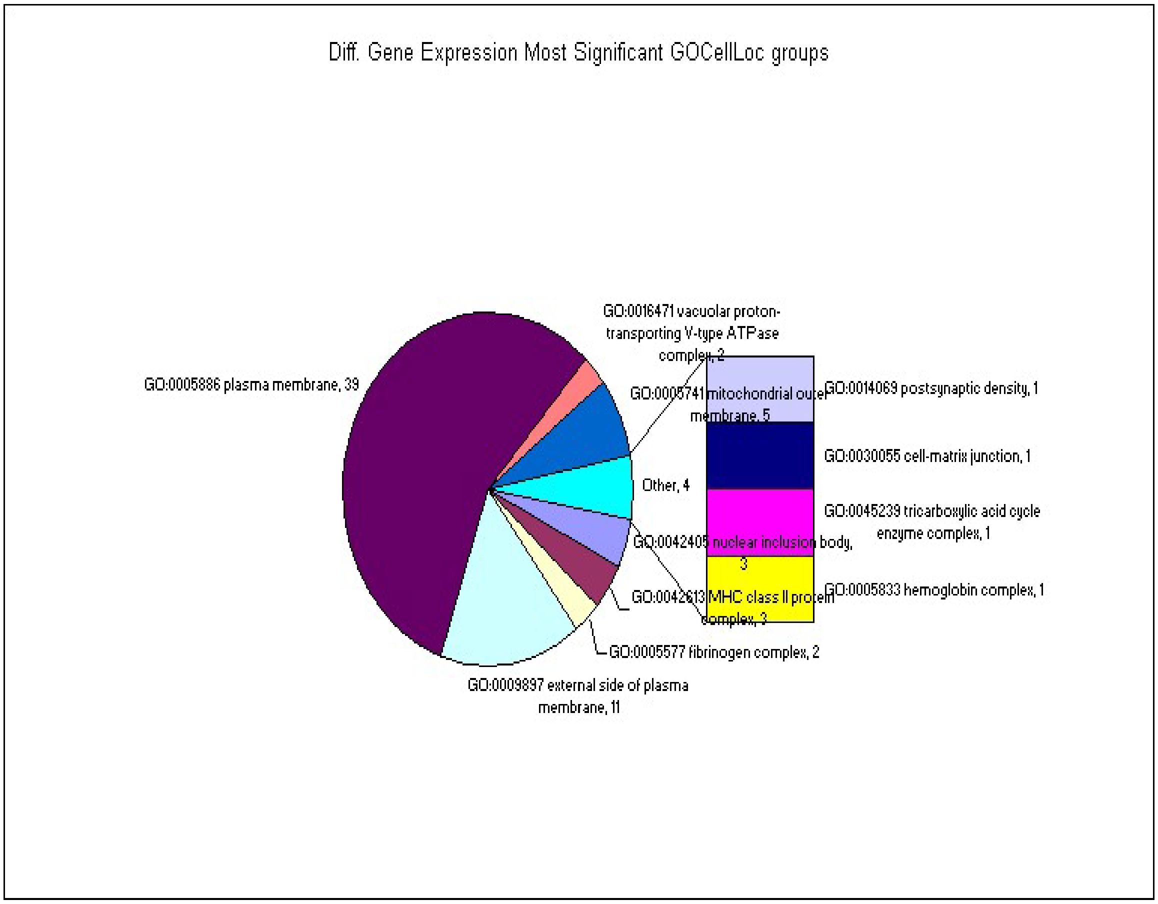

Supplementary Figure 1. The GOCellLoc gene classifications that were significantly overrepresented in the set of differentially spliced or expressed genes.
